# Graphitized Biochar Derived from Agricultural Wastes Enhances Methanogenesis via Conductivity‐Driven Direct Interspecies Electron Transfer

**DOI:** 10.1002/advs.202508739

**Published:** 2025-08-04

**Authors:** Caiyun Yang, Zhen Liu, Weiguo Liu, Yuxin Qiu, Shuai Zhang, Xinke Zhang, Mengyi Wang, Heng Wu, Hongyi Lyu, Jinzhi Huang, Jia Liu, Yirong Wang, Siying He, Dongze Gu, Xiaohui Guo, Xuanmin Yang, Teng Xie, Heyu Chen, Yiqing Yao

**Affiliations:** ^1^ College of Life Science Northwest A&F University Yangling Shaanxi 712100 P. R. China; ^2^ College of Mechanical and Electronic Engineering Northwest A&F University Yangling Shaanxi 712100 P. R. China; ^3^ Interdisciplinary Research Center for Biomass Energy and Materials (IRC‐BEM) Northwest A&F University Yangling Shaanxi 712100 P. R. China; ^4^ Northwest A&F University Shenzhen Research Institute Shenzhen 518000 P. R. China; ^5^ College of Forestry Northwest A&F University Yangling Shaanxi 712100 P. R. China; ^6^ College of Food Engineering & Nutritional Science SNNU Shaanxi Normal University Xian Shaanxi 710119 P. R. China; ^7^ State Key Laboratory of Urtan Water Resource and Environment School of Environment Harbin Institute of Technology Harbin 150090 P. R. China; ^8^ QiCheng Suspension Technology Co,.Ltd Xian 710086 P. R. China

**Keywords:** acetoclastic methanogenesis, anaerobic digestion, biochar conductivity, direct interspecies electron transfer (DIET), graphitized carbon

## Abstract

Biochar has emerged as a promising conductor for facilitating direct interspecies electron transfer (DIET) in anaerobic digestion (AD), yet the mechanisms linking its structural features to methanogenic performance remain unclear. Here, how feedstock type and pyrolysis conditions modulate biochar conductivity and consequently shape methanogenic pathways is investigated. Using straw, wood, and nutshell‐derived biochars, nutshell biochar pyrolyzed at 550 °C(CC550) is demonstrated to achieve the highest methane yield, enhancing production by 59% compared to the control and outperforming straw and wood‐based biochars by 12% and 5% respectively. Graphitization analysis confirms that high electrical conductivity is key to accelerating methanogenesis. Metagenomic profiling reveals that CC550 enriches cellulose‐degrading bacteria and DIET‐associated taxa, while upregulating genes related to pili and cytochrome c expression, promoting acetoclastic methanogenesis through enhanced electron flow. These findings highlight the role of graphitic biochar as a metabolic modulator in AD and offer insights for engineering carbon materials to optimize bioenergy recovery from organic waste.

## Graphical abstract

This work clarifies how feedstocks and their structural features promote methanogenesis, and identifies the key regulatory factors, reducing pollution to the global environment, and also helps to alleviate the energy crisis. This is the first study to systematically link feedstock lignin content, pyrolysis conditions, biochar graphitization, and DIET efficiency in AD systems.

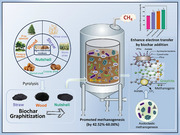

## 1 Introduction

Forestry and agricultural residues, including crop straw, forestry trimmings, and animal manure, represent abundant organic waste streams generated globally. With over 2 gigatonnes (Gt) of municipal and agricultural waste produced annually worldwide, biomass residues account for ≈11% of this total.^[^ ^1^ ^]^ In developing countries, 65–78% of these residues are either left to decompose or openly burned. Potentially releasing 1.2–1.5 Gt of CO_2_ annually by 2025, ≈5% of global anthropogenic emissions (IPCC, 2023; FAO, 2022). Anaerobic digestion (AD) effectively degrades this waste and converts it into clean energy, making it a crucial strategy for achieving circular agriculture.^[^ ^2^ ^]^ However, due to the highly cross‐linked structure and crystallinity of lignocellulosic materials, such as crop straw, make the hydrolysis of cellulose and hemicellulose challenging, leading to low conversion rates.^[^ ^3^, ^4^ ^]^ Additionally, its high carbon nitrogen ratio (C/N) tends to accumulate excessive volatile fatty acids (VFAs), leading to hydrolysis inhibition and low gas production rates.^[^ ^5^ ^]^

Biochar has been widely demonstrated to enhance methane production during AD due to its physicochemical properties, including high electrical conductivity, abundant oxygen‐containing functional groups, developed porosity, and large surface area.^[^ ^6^ ^]^ Recent studies have identified feedstock composition and pyrolysis temperature as two critical parameters that govern the methanogenic efficiency of biochar.^[^ ^7^ ^]^ Notably, the physicochemical characteristics of biochar are intrinsically determined by the structural features of its precursor materials. Cellulosic and hemicellulosic‐rich feedstocks typically yield biochars with lower fixed carbon content and aromaticity but higher concentrations of oxygen‐containing functional groups, whereas lignin‐derived biochars exhibit significantly enhanced porosity, surface area, aromaticity, stability, and fixed carbon content.^[^ ^8^ ^]^ Recent investigations further reveal synergistic effects between feedstock selection and pyrolysis conditions on biochar properties, which can be attributed to the distinct thermal decomposition behaviors and chemical structures of cellulose, hemicellulose, and lignin.^[^ ^9^ ^]^ For instance, Zheng et al.^[^ ^10^, ^11^ ^]^ systematically characterized the pyrolysis regimes of untreated commercial lignocellulosic components, demonstrating that cellulose, hemicellulose, and lignin undergo optimal carbonization within temperature ranges of 220–315 °C, 315–400 °C, and 350–500 °C, respectively, thereby facilitating biochar formation.

The electron transfer dynamics and microbial immobilization mechanisms of biochar in AD have been extensively investigated.^[^ ^12^ ^]^ Valentin et al.^[^ ^13^ ^]^ discovered that highly conductive biochar acts as an electron conduit, enhancing direct interspecies electron transfer (DIET) between exoelectrogenic bacteria such as *Geobacter* and methanogenic archaea such as *Methanosaeta*. Gao et al.^[^ ^14^ ^]^ suggested that the redox activity of the surface functional groups of biochar primarily enhances the hydrolysis‐acidification phase. Notably, He et al.^[^ ^15^ ^]^ reported a 2–3 fold increase in methanogenic rates when using biochar with graphite content exceeding 60%, implying that conductivity‐dependent DIET mechanisms predominate in later AD stages. The conductive properties of biochar are intrinsically linked to its oxygen‐containing functional groups and graphitized carbon structures.^[^ ^16^ ^]^ While functional groups contribute to electron transport, studies indicate that graphitization becomes the dominant driver of conductivity under high‐temperature pyrolysis (>500 °C).^[^ ^17^ ^]^ Yuan et al.^[^ ^18^ ^]^ observed that higher lignin content in feedstocks contributes to a well‐developed porous structure and larger specific surface area, promoting methanogen colonization. Additionally, Cheng et al.^[^ ^19^ ^]^ found that increased graphitization leads to stable conjugated π‐bond systems within biochar, enhancing conductivity. Therefore, we hypothesize that the graphitized structures of high lignin feedstocks may lead to methanogenic enhancement.

This study hypothesizes that variations in lignin content among straw, wood, and nutshell biochar feedstocks lead to differences in their graphitization levels, thereby affecting methane yields during AD. To verify this hypothesis, we applied these three biochar types to a sequential batch anaerobic digestion (SBAD) using maize straw. 16S rRNA amplicon sequencing and metagenomic were used to analyze the changes in performance, microbial community structure, and metabolic pathways during AD. Subsequently, the mechanism by which biochar enhances AD methane production was investigated. The methane yield results were then validated by modified biochar addition with a higher degree of graphitization, and a new enhancement strategy is proposed to improve energy conversion efficiency. It was anticipated that this study would provide new insights into the role of biochar in methanogenesis and theoretical support for its industrial production.

## 2 Results

### 2.1 Biochar Demonstrate Optimal Methanogenesis Efficiency by Alleviating Acid Inhibition

**Figure 1:**
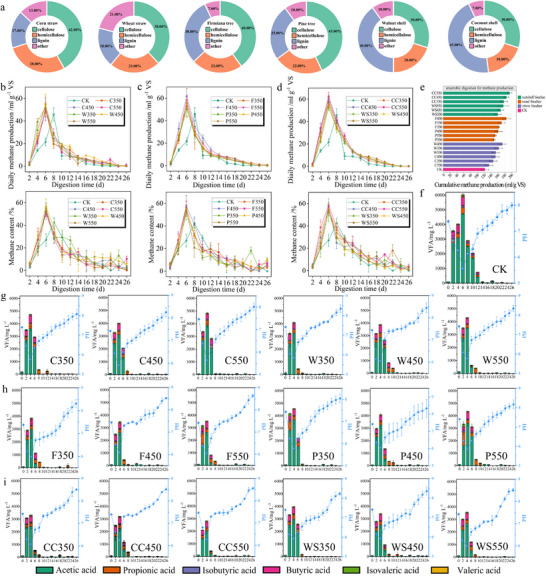
The impact of biochar addition on methane production and volatile fatty acids in AD. a) The contents of hemicellulose, cellulose, and lignin in different biochar feedstocks.^[^ ^48^, ^49^, ^50^, ^51^, ^52^ ^]^ b–d) Daily methane production, methane content for straw, wood, nutshell biochar. e) Cumulative methane production on the 26th day for straw, wood, and nutshell biochar. f–i) PH and VFA changes over time for CK, straw, wood, and nutshell biochar. *p* ≤ 0.05, one‐way ANOVA, Dunnett's test.

Nutshell biochar feedstocks exhibited significantly higher lignin content (43.3 ± 2.1 wt.%) compared to wood (28.2 ± 1.8 wt.%) and straw (17.5 ± 1.2 wt.%) sources, representing 1.53‐fold and 2.38‐fold increases (**Figure** **1**a). Based on the changes of methane production, VFAs, and pH (Figure 1), nutshell biochar (CC550) had the highest methanogenesis efficiency with a daily yield of 62.5 mL g^−1^ VS and a 59.3% cumulative increase, outperforming the two other kinds of biochar (Figure 1d,e). Wood biochar (F450) followed (61.8 mL g^−1^ VS) (Figure 1c,e), while straw biochar (W450) had the lowest yield (55.1 mL g^−1^ VS) (Figure 1b,e). The lignin content in the three feedstocks demonstrated significant positive correlation with methane production enhancement (R^2^ = 0.91, p<0.5) (Figure S1, Supporting Information). Specifically, biochar feedstocks containing lignin (17.5–43.3 wt.%) exhibited a strong positive correlation with cumulative methane yields (55.1–62.5 mL g^−1^ VS) (Figure 1a,e; Figure S1, Supporting Information). All treatments (straw, wood, and nutshell) completed acidification‐buffer regulation 2 days earlier than the control. By Day 4, CC550 had the lowest VFA concentration and the weakest acid inhibition^[^ ^20^ ^]^ (Figure 1i). In contrast, the control reached a peak VFAs concentration of 6.03 g L^−1^ on Day 6 (pH 5.55) (Figure 1f), while CC550 maintained a stable pH of 6.07 (+0.08). By Day 8, acetic and butyric acids in CC550 were nearly depleted (Figure 1i), whereas over 50% of acetic acid remained in the control (Figure 1f), significantly reducing acidification pressure.^[^ ^21^ ^]^ Methane yield peaked on Day 6 in the treatment groups, whereas the control peaked on Day 8, shortening the lag phase by 2 days (Figure 1d). The TS:VS degradation rate correlated positively with methanogenesis efficiency (nutshell > wood > straw) (Table S5, Supporting Information), indicating that the conversion rate of substrate and the path of product generation were optimized synchronously.

### 2.2 Structural Graphitization Enhances Methanogenic Efficiency in Biochar Systems

**Figure 2:**
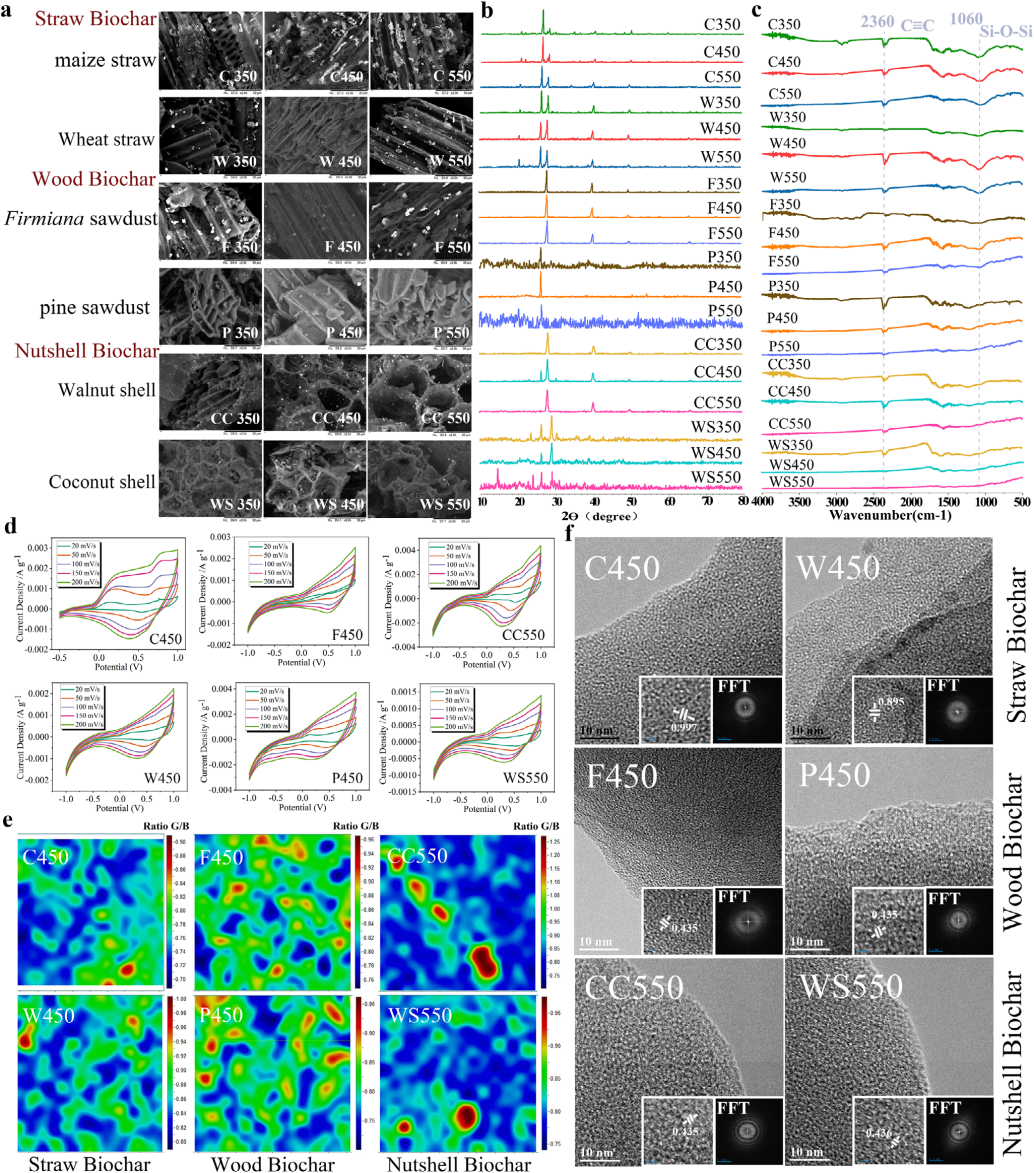
Physiochemical Properties of Biochar. a) SEM of biochar from maize straw, wheat straw, *Firmiana*, pine tree, walnut shells, and coconut shells pyrolyzed at 350, 450, 550 °C (×2000 magnification). b,c) XRD, FTIR spectra of biochar from the same feedstocks at the indicated temperatures. d) CV curves of biochar at different scanning speeds. e) Raman mapping of biochar. f) TEM characterization of biochar crystalline graphitized.

XRD analysis revealed a weak diffraction peak at 2θ = 22°, indicating the presence of cellulose and hemicellulose. At 350 °C, a broad peak at 28° appeared, while at 450 °C, the 002 diffraction peak of graphitized microcrystalline carbon emerged at 26.6°, indicative of high‐temperature conversion of amorphous carbon into graphitic structures.^[^ ^22^ ^]^ The 002 peak intensity of straw biochar (W450) was only 42% of that for nutshell biochar. Although wood biochar (F450) had a weak graphitization peak at 450 °C, it lacked thermal stability (**Figure** **2**b). At 550 °C, nutshell biochar retained a 5.4% micropore increase, while straw and wood biochar suffered pore collapse rates of 14.8% and 69.6%, respectively. Consequently, the specific surface area of nutshell biochar increased to 17.1 m^2^ g^−1^, surpassing straw (15.0 m^2^ g^−1^) and wood biochar (10.2 m^2^ g^−1^) (Figure 2a; Table S6, Supporting Information). Nutshell biochar had greater specific surface area, pore volume, and mesopore size, enhancing spatial compatibility with methanogenic archaea.^[^ ^23^, ^24^ ^]^ Its anti‐collapse ability and pore adaptability were evident. FTIR analysis at 1600 cm^−1^ detected a strong C═O stretching vibration, indicating high quinone abundance. The high crosslinking degree of nutshell‐derived lignin inhibited the removal of ─CH_2_ (2800 cm^−1^) and ─OH (3400 cm^−1^) functional groups, whereas straw lignin decomposition decreased oxygen‐containing groups by over 65% (Figure 2c). Thus, the 59.3%‐increase in methane production with nutshell biochar was attributed to its graphitic microcrystalline carbon, collapse resistant pores, and quinone dominated electron shuttling derived from high‐temperature lignin based carbon networks.^[^ ^25^ ^]^

At 450 °C, C450 had a larger CV curve enclosed area than other biochar, indicating a stronger electron exchange capacity principally relying on self oxidation reduction reaction. In contrast, the weaker oxidation peak current of other biochar could be related to increased pyrolysis temperature and decreased oxygen containing functional groups. Nutshell biochar had a prominent reduction peak current, by now, electron transport mainly relied on its intrinsic conductivity (Figure 2d). Extensive blue regions within nutshell biochar, indicating higher carbon ordering and a more compact atomic structure^[^ ^26^ ^]^ (Figure 2e). This was further confirmed by the electron diffraction rings in Fast Fourier Transform(FFT) (Figure 2f), which indicated a transition from amorphous to graphitic carbon, consistent with the 002 diffraction peak enhancement in XRD.^[^ ^27^ ^]^ By comparison, straw biochar had an ID/IG ratio of 0.7‐0.83, containing amorphous carbon (green regions), while wood biochar contained both amorphous carbon (green) and partially graphitization regions (red) (Figure 2e), indicating that nutshell biochar had a significantly higher degree of graphitization than wood and straw biochars. Additionally, CC550 had a stronger G‐band intensity, further confirming its more developed graphitic structure (Figure S3, Supporting Information). Notably, the lower the ID/IG of the biochar added to AD, the more methane was produced (R^2^ = 0.81, p<0.05). We hypothesis that the degree of graphitization is the leading factor influencing AD. With its highly graphitized structure, nutshell biochar achieved the most significant improvement in methane yield.

### 2.3 Biochar Addition on Dynamic Succession and Functional Regulation of Microbial Communities

**Figure 3:**
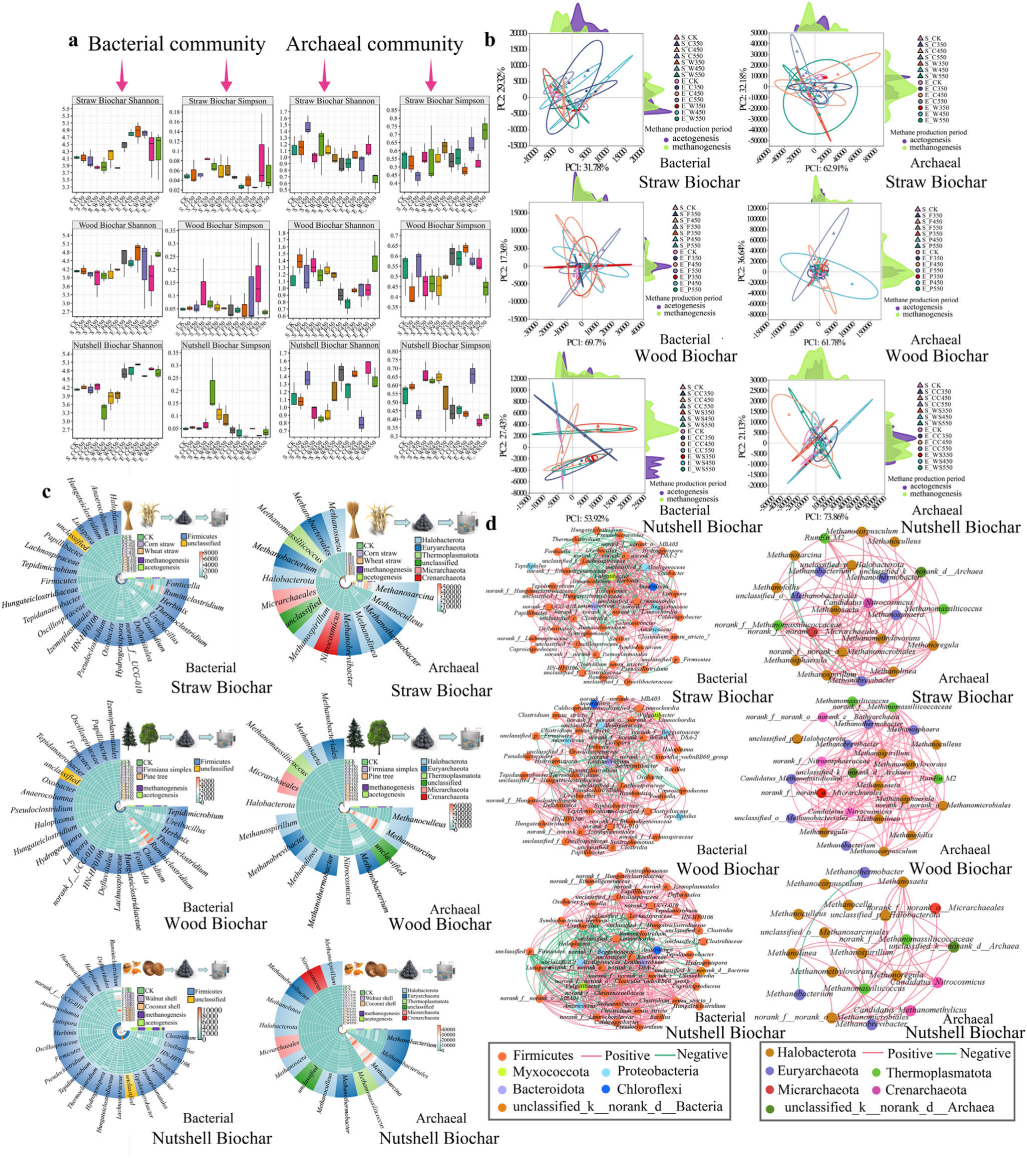
The diversity index, microbial community composition, PCA analytical, and co‐occurrence network involved in AD with biochar addition. a) The diversity index using the Shannon and Simpson index. *t*‐test was used to evaluate the communities differences between AD during the peak and end stages. b) PCA analysis reflected the discrepancy in the microbial community. The green represents the acetogenesis stage, the purple denotes the methanogenesis stage. c) The microbial community composition in AD. Samples were clustered using hierarchical clustering with complete linkage based on euclidean distance. d) The co‐occurrence network of microbe. A co‐occurrence network was constructed based on the 30 most abundant species at the genus level, utilizing the Spearman coefficient as the distance metric to analyze correlations between species and identify key taxa. Only Spearman coefficients with an absolute value ≥ 0.5 and *p* < 0.05 were considered significant and visualized in the network. The size of the nodes represents the relative abundance of the microbe. And modularized according to relative abundance size, with stronger interspecies interaction within the same module.

The effective sequence coverage of the samples exceeded 98% (OTU similarity 0.97), ensuring microbial community authenticity. At the end of the gas production stage, bacterial richness was significantly higher than at the peak stage. During the transition from peak to the end of methane production, the Shannon index increased while the Simpson index decreased (**Figure** **3**a), indicating that the community changed from a single dominant species to succession with multiple coexisting species. Nutshell biochar at 550 °C had the closest clustering in the PCA, whereas straw and wood biochar at 450 °C had the smallest sample distance. Sample variation was more pronounced, particularly in the final stage of the archaeal communities (Figure 3b).

Biochar addition significantly refactored the functional microbial community during AD. During the hydrolysis‐acidification stage, bacterial metabolism was dominated by Firmicutes (70% to 90%), along with Proteobacteria, Bacteroidetes, Chloroflexi, and Myxococcota (Figure 3c). Firmicutes had a dynamic shift, peaking at 97.2 ± 3.5% at peak gas production, then decreasing to 76.7 ± 2.8% by the end. Key genus (*Ruminiclostridium*, *Herbinix*, and *Ureibacillus*) had stronger synergistic interactions at the peak stage (co‐occurrence network edge weight > 0.82). In contrast, *Hydrogenispora* appeared to have an antagonistic relationship with *Ruminiclostridium* and *Herbinix* (r = −0.67, p < 0.05) and increased by 39.0% to 85.7% under high temperatures (Figure 3d). During the methanogenic phase, biochar enriched communities were dominated by acetoclastic *Methanosarcina* (72.9 ± 0.2%) and hydrogenotrophic *Methanobacterium* (16.5 ± 0.2%).^[^ ^28^ ^]^ Compared to straw and wood biochar, nutshell biochar had increased H_2_/CO_2_‐utilizing *Methanothermobacter* (9.5 ± 0.1%) and *Methanoculleus* (7.2 ± 0.1%) by the final stage (Figure S4, Supporting Information). *Methanosarcina* correlated positively with *Methanomassiliicoccus* but negatively with *Methanothermobacter* (Figure 3d). The porous structure (BET > 17 m^2^ g^−1^) facilitated *Methanosarcina* aggregation. In the control (without biochar), microbial abundance changed by only 0.01% to 3.5% from peak to the final stage, whereas in biochar treated groups, microbial succession reached 0.01% to 19.8% (*p* < 0.001) (Figure S4, Supporting Information), confirming biochar targeted regulatory role in microbial metabolism.

### 2.4 Methanogenesis and DIET Pathway in Aerobic Digestion with Biochar Addition

**Figure 4:**
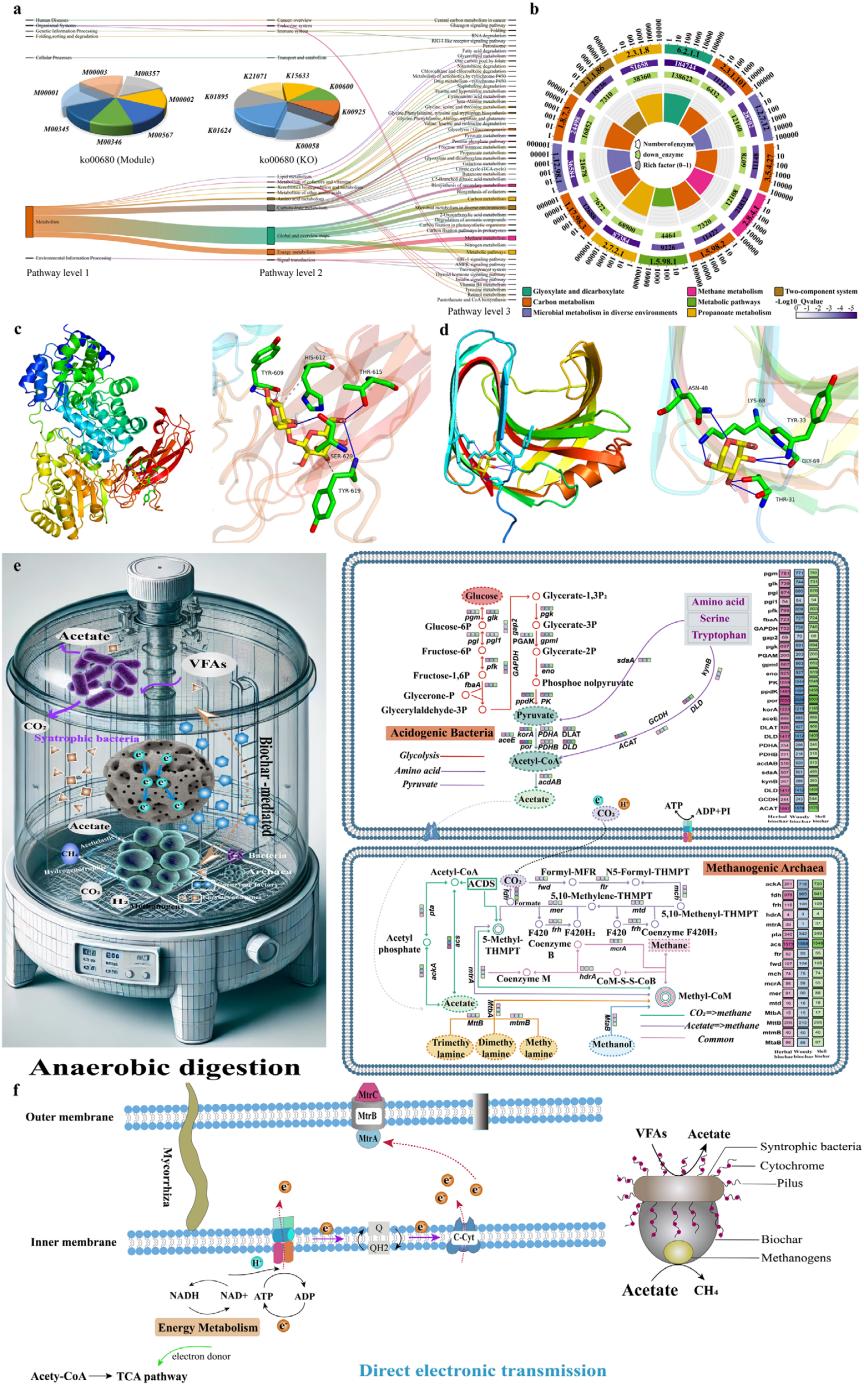
Methane metabolism in AD difference by biochar addition. a) Abundance of different samples at Pathway (level 1/2/3)/Module/KO. b) Optimal molecular docking model of cellulose and *bglX*, as well as a schematic diagram of the interaction force. c) Optimal molecular docking model of hemicellulose and *xynA*, as well as a schematic diagram of the interaction force. d) Enzyme activities of the top 13 significantly expressed enzymes in methane metabolism. e) Gene expression in the methane metabolism pathway. f) Biochar promoted the DIET pathway.

After quality control filtering (removal of low‐quality and N‐containing sequences), a functional database of 852986 non‐redundant genes was constructed. KEGG annotation showed that biochar addition significantly regulated gene abundance in metabolic pathways (p < 0.05), with the most affected pathways including carbon fixation (ko00720), glycolysis/pyruvate metabolism (KO00010 & KO00620), serine cycle (KO00260), and methane metabolism (ko00680). Notably, nutshell biochar had the greater effect on methane metabolism (1.03 times), consistent with its methane yield advantage (+59.8%) (**Figure** **4**a). Co‐occurrence network analysis revealed a strong positive correlation between *Symbiobacterium*, and *Methanosarcina* and *Methanoculleus*, indicating that *Symbiobacterium* may mediate DIET via conductive pili.^[^ ^29^ ^]^ It also had metabolic site competition with acidogenic bacteria *Oscillospiraceae* and *Clostridium*. Additionally, the weak positive correlation between *Methanoculleus*, *Methanobacterium* and *Clostridium* indicates a potential acetate cross‐feeding interaction^[^ ^30^ ^]^ (Figure S5e, Supporting Information). In the nutshell biochar group, *Methanosarcina* was the dominant methanogen, with an increased abundance of the *acs* gene (Figure 4e), while *Methanobacterium* and *Methanoculleus* functioned as secondary species, facilitating electron triage via cytochrome c oxidase (EC 1.9.3.1) at levels 1.7–3.2 times higher than in the wood and straw biochar treatments^[^ ^31^ ^]^ (Figure S5e, Supporting Information). At 550 °C, genes encoding acetate kinase (EC 2.7.2.1) and phosphate acetyltransferase (EC 2.3.1.8) were upregulated, promoting the *Methanosarcina* mediated *pta*‐*ackA* pathway in a reversible manner.^[^ ^32^ ^]^ Acetate was incorporated into the TCA cycle via acetyl‐CoA synthetase (EC 6.2.1.1) (Figure 4b). In addition, conductive pili genes (*mtrA*, *mtrB*, and *mtrC* increased 1.25, 1.51, and 2.36 times, respectively) and cytochrome genes (*petA*, *petB*, and *cycA* increased 1.01, 2.08, and 1.85 times, repetitively) provided strong genetic support for DIET (Figure S5d, Supporting Information). DIET demonstrates higher electron transfer efficiency compared to interspecies hydrogen transfer (IHT), bypassing the thermodynamic constraints associated with hydrogenotrophic pathways. This enhanced efficiency is evidenced by DIET's 8.57 fold faster electron transfer kinetics relative to IHT mediated processes.^[^ ^33^ ^]^

The reconstructed methanogenesis and carbon assimilation pathways primarily supported acetoclastic methanogenesis (56.3% contribution), alongside the acetyl‐CoA, serine, and ribulose phosphate pathways, whereas the methylamine/formate pathway remained at 18.7%. The serine pathway (ko00260) facilitated C1 transfer for carbon assimilation via tetrahydrofolate (Figure 4a). Under anaerobic conditions, the normal pentose phosphate pathway was replaced by sedoheptulose‐1,7‐bisphosphate, where S7P was phosphorylated to S7BP via PPi‐PFK, then cleaved into DHAP (C3) and E4P, enabling carbon backbone reconstruction (Figure 4e). In a nutshell biochar (log2FC > 0, p < 0.05), polysaccharide hydrolysis genes (*xynA* and *bglX*) were significantly upregulated, confirming their key contribution. For lignocellulose hydrolysis, enzymes encoded by *bglX* and *xynA* facilitated molecular docking with cellulose and hemicellulose, have optimal binding energies of −6.7 and −5.5 kcal mol^−1^, respectively (Tables S8 and S9, Supporting Information). These enzymes stabilized cellulose and hemicellulose degradation through hydrogen bonding and hydrophobic interactions (Figure 4c). The mesoporous structure of nutshell biochar enhanced *Herbinix* carbon metabolism and accelerated the expression of EC 2.7.2.1 encoded genes.^[^ ^34^ ^]^ In contrast, wood biochar promoted acetate synthesis and hydrogen production by *unclassified_f_Oscillospiraceae*,^[^ ^35^ ^]^ but its electron transfer associated cytochrome c abundance was only 46% of that in nutshell biochar (Figure S6a, Supporting Information). By facilitating metabolic interactions between hydrolytic bacteria and methanogens, nutshell biochar shifted thermodynamic equilibrium toward acetoclastic methanogenesis. Additionally, biochar significantly enriched DIET associated microorganisms (Figure 4f), further confirming its role in enhancing electron transfer through DIET induction.

### 2.5 Verifying the Degree of Biochar Graphitization Enhances Methanogenesis

**Figure 5:**
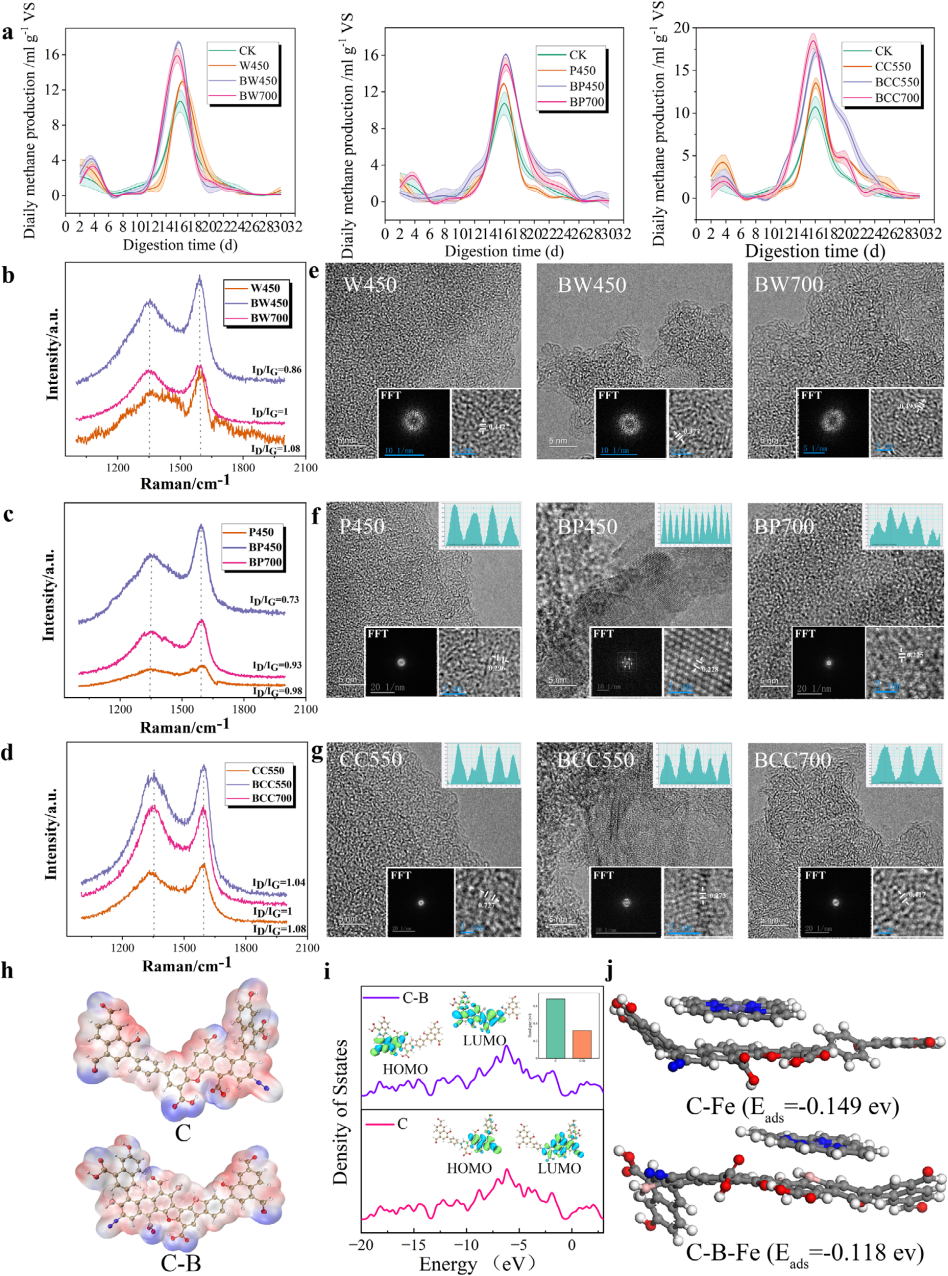
Methanogenesis and Conductive characterization of modified biochar. a) Daily methane production for modified biochar. b–d) Raman spectrum of modified biochar. e–g) HRTEM of modified biochar crystalline graphitic. h) ESP of biochar and modified biochar. i) DOS of biochar and modified biochar. j) The adsorption energy of cytochrome C by biochar and modified biochar.

**Figure 6:**
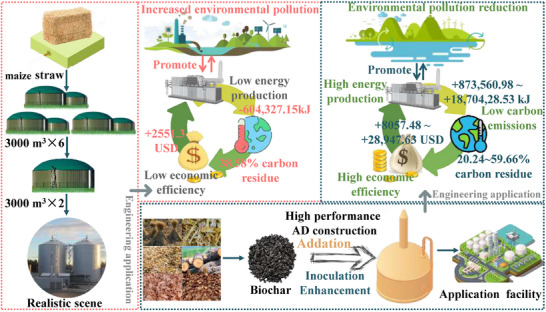
Economic and environmental benefits enhanced AD compared to the Cangzhou biogas plant.

The addition of boron‐modified biochar enhances the graphitization degree, aiming to verify that the graphitized structure indeed promotes methanogenesis in AD.^[^ ^36^ ^]^ BW450 and BP450 gave the highest cumulative methane yields (46 ± 1.1 and 51 ± 0.9 mL g^−1^ VS), which were 6.7% to 13.2% higher than BW700 and BP700 (**Figure** **5**a). For nutshell biochar, BCC700 reached a peak daily methane production of 18.0 mL g^−1^ VS, which was 1.7 times higher than BCC550 (*p* = 0.003, *t*‐test) (Figure S6a, Supporting Information). Alkalinity contribution revealed universally weak acid‐buffering capacities across biochars, graphitized biochar demonstrated markedly enhanced electron transport capability compared to non‐graphitized biochar, where methane yields showed significant positive correlation with electrical conductivity but no association with β‐alkalinity, indicating conductivity as the dominant regulatory factor (Figure 5i; Figure S6b, Supporting Information). HRTEM demonstrated that BW450/BP450 (d002 spacing = 0.347 nm) and BCC550 (ID/IG ratio = 1.04) (Figure 6b–d) exhibited enhanced graphitization corresponding to improved methanogenic. Notably, a negative correlation between pH and electrical conductivity (EC) was observed during anaerobic digestion (Figure S6c–e, Supporting Information), potentially linked to H⁺‐mediated shifts in ionic strength. Biochar conductivity was governed by intrinsic characteristics including carbon framework graphitization, ash composition, and pore architecture.^[^ ^37^, ^38^ ^]^ BCC700 displayed remarkably developed deep pore structures (Figure 6d), which could provide optimal microhabitats for electroactive methanogens such as *Methanosarcina*.^[^ ^39^ ^]^ The reduced heme adsorption capacity coupled with enhanced conductivity suggests a mechanistic transition from conventional “enzyme adsorption” to “electron transfer mediation”, prioritizing direct interspecies electron transfer (DIET) over enzymatic interactions (Figure 5j). Thus, biochar boosts methane production through a graphitized carbon framework improves electron transfer, and a hierarchical pore structure shortens the electron transfer distance between microorganisms.

### 2.6 Application Implications

The global warming potential (GWP) of various processes and scenarios was used to evaluate the sustainability of AD. Compared to the control, nutshell biochar had 59.7% less GWP and would generate significantly lower emissions compared to other biochar (Figure 6). Energy balance calculations revealed a 10.6% increase in net energy utilization with nutshell biochar addition, whereas the control improved by only 3.3% (Figure S3c, Supporting Information). Equipment costs dictate the economic payback period. Compared to the control, using coconut nutshell biochar provided an elevenfold increase in profit and decreased the investment payback period to 34 days (Figure 6). The addition of biochar overcame the problem of low gas production. This would mean that the annual biogas production of 24000 m^3^ in Cangzhou Gengguantun biogas plant could have a 333‐fold increase in production with biochar‐enhanced CH_4_ production increased. In conclusion, add biochar significantly enhances efficiency, generates substantial profits, and possesses extensive application potential.

## 3 Discussion

The challenges of energy crises and environmental pollution pose significant threats to global economic development. Straw as a lignocellulose‐rich feedstock for sustainable bioenergy production.^[^ ^40^ ^]^ Anaerobic digestion‐based circular economy offers a viable strategy to decouple economic growth from resource consumption. While we have extensively validated the methane enhancing of biochar in AD, the relationships between biochar feedstock and pyrolysis temperature and methanogenic remain poorly defined. Our findings further revealed that nutshell biochar exhibited the highest methane yield (194.5 mL·g^−1^ VS), followed by wood (185.1 mL·g^−1^ VS) and straw biochar (173.6 mL·g^−1^ VS). Importantly, lignin content showed a strong positive correlation with methane yield enhancement (R^2^ = 0.91, p<0.05), suggesting that the differences in structure and pyrolysis characteristics among cellulose, hemicellulose, and lignin in feedstock may be the cause of this phenomenon. Previous studies have demonstrated that biochar's enhancement of methanogenesis in AD is attributed to conductivity, functional groups, and porosity. The porosity serves as microbial niches, Functional groups promote the transformation of intermediate products, and enhance electron transfer for increasing methanogenesis.^[^ ^41^ ^]^ Notably, both the functional groups and graphitic structure of biochar can affect electrical conductivity, but its contribution to methanogenesis in AD remains unclear. While moderate graphitization combined with functional groups can synergistically stimulate interspecies electron transfer (IET), the observed weak oxidation peak currents in CV analyses indicate that electron transfer predominantly relies on biochar's intrinsic conductivity rather than reversible redox reactions of surface functional groups. Furthermore, our findings revealed a significant inverse correlation between the lower the ID/IG, the higher the methane production (R^2^ = 0.81, *p* < 0.05). This suggests that graphitic structures in biochar may serve as a critical determinant for methanogenic performance. This graphitic layered structure not only provides a good conductive network for the migration of electrons within the plane,^[^ ^42^ ^]^ but also further promotes the electron exchange between microorganisms, ultimately enhancing the efficiency of methanogenesis.

Furthermore, we performed 16S rRNA gene amplicon sequencing to characterize the microbial communities involved methanogenesis in AD. The results show that Biochar selective enrichment of microorganisms related to direct interspecific electron transfer (DIET), such as Bacteroidetes, Chloroflexi, and Proteobacteria, *Methanosarcina*, *Methanosaeta*, *Methanobacterium* and *Methanothrix* maintained the dynamic balance of the acid and methane production processes. Further metagenomic results indicated that after reconstructing the methane generation and carbon assimilation pathways, it was found that the enzyme genes of the acetoclastic methanogenesis, the acetyl Co‐A pathway, the serine pathway, and the Ribuloae‐P pathway mainly existed in the system. Among them, the abundance of enzyme genes (*pta*, *ackA* and *acs*) of the acetoclastic methanogenesis was significantly increased. When adding highly conductive graphite, it also promotes the DIET to increase methane production by enhancing the acetoclastic methanogenesis.^[^ ^43^ ^]^ These findings suggest that microorganisms can achieve stable CH_4_ production by accelerating acetic acid degradation, regulating acetoclastic methanogenesis, through glycolysis, pyruvate metabolism pathways, and a highly conductive DIET pathway. Notably, this process proved more efficient and environmentally friendly than standard AD systems. Compared to the control, nutshell biochar decreased GWP emissions by 60%. In addition, the current annual 24000 m^3^ biogas production at Cangzhou Gengguantun biogas plant could have a 333‐fold increase in CH_4_ yields.

To our knowledge, this is the first to clearly identify the key factor driving biochar enhanced methanogenesis in AD. The findings not only mitigate hydrolysis‐acidification inhibition and optimize the digestion process but also offer significant advantages in pollution reduction and economic benefits. Notably, this approach has demonstrated strong scalability and substantial economic potential, making it particularly valuable for facilities engaged in agricultural waste resource utilization worldwide.

## 4 Experimental Section

### Anaerobic Digestion with Biochar Addition

The maize straw used was sourced from a farm in the Yangling Demonstration Zone. After air drying, it was cut into 3–5 cm pieces, ground, and passed through a 0.9‐mm sieved before being sealed and stored. Inoculum was obtained from the secondary treatment sludge of the Yangling demonstration area sewage treatment plant, and domestication under laboratory conditions was used previously. Digestion substrates (maize straw), inoculum (laboratory domesticated activated sludge), and three types of biochar (straw, wood, and nutshell) were added to a 500 mL fermenter with an effective volume of 350 mL. The addition rate of the digestion substrate was 5% of the dry substrate mass, while the inoculum addition rate was 30% (v/v). Based on the physicochemical parameters of the substrate and inoculum (Table S2, Supporting Information), the amount of biochar added was 5% of the dry mass of the digestion substrate. After all materials were added, these were mixed thoroughly with water and purged with nitrogen for 1 min before sealing. The control did not include biochar, while the blank contained neither biochar nor maize straw to eliminate the influence of the inoculum on gas production. After assembling 60 reactors, the SBAD was conducted at a constant temperature of 55 ± 1 °C. Gas was collected every 2 days using 2 L aluminum foil gas collection bags, and VFAs composition and pH of the AD determined. AD was deemed to have finished when the quantity of gas fell below 20 mL and the methane content was less than 10%. Therefore, the digestion time was 26 days.

### Biochar Preparation and Characterization

The feedstocks for biochar preparation include maize straw, wheat straw, pine sawdust, *Firmiana* sawdust, walnut shells, and coconut shells. Maize and wheat straws were sourced from a farm in the Yangling Demonstration Zone, Shaanxi Province; pine and *Firmiana* sawdust were obtained from a timber factory in Xi'an, Shaanxi Province; walnut shells were bought at a market near Northwest A&F University; and coconut shells were purchased in Wenchang City, Hainan Province. After washing the feedstocks with deionized water, they were drained over a 0.42‐mm sieve. Subsequently, they were dried at a constant temperature of 75 °C for 48 h. The chamber of the tube furnace used to make the biochar was first evacuated and then filled with nitrogen. After placing the feedstocks in the furnace, the temperature was increased at a rate of 10 °C min^−1^ to 350, 450, and 550 °C, respectively, for pyrolysis of straw, wood, and nutshell substrates. Following pyrolysis, the biochar was washed with deionized water to remove surface ash. After drying, the biochar was ground to a particle size of 0.30–0.45 mm and stored the biochar according to the type of feedstocks as given in Table S1 (Supporting Information).

The surface appearance of the biochar was observed with a scanning electron microscope (SEM).^[^ ^44^ ^]^ The specific surface area was measured using a specific surface area analyzer using both the Brunauer‐Emmett‐Teller (BET) and Langmuir equations. Aperture analyzer was used to measure the size of micro‐, meso‐, and macropores. The static gauging method was used for the determination of isothermal nitrogen adsorption and desorption. The crystal structure of the biochar was characterized using X‐ray diffractometer (XRD), and the functional groups were analyzed using Fourier transformed infrared spectrum (FTIR).^[^ ^45^ ^]^

A saturated calomel electrode was used as the reference electrode, and the biochar electrode and counter electrode as the working electrodes to measure the cyclic voltammetric (CV) curves of the biochar electrode.^[^ ^12^ ^]^ Raman spectroscopy and mapping were used to analyze the biochar surface graphitization degree, and high‐resolution transmission electron microscopy (HRTEM) to observe the crystalline graphite products in the biochar. The wave function analysis methods adopted included electrostatic potential (ESP), density‐of‐state (DOS), the adsorption energy of cytochrome C by biochar structure. The above analysis was performed using Castep, and Gussess 1.3 was used to visualize the analysis results.

The study used a cradle‐to‐grave life cycle assessment (LCA) model to analyze carbon emissions in different scenarios, using 1 GJ of methane production as the functional unit. The modeling covered residue collection, transportation, preprocessing, and methane production. The residues were baled, loaded onto a forage wagon, and transported by truck over an average distance of 50 km. At the plant, the residues were chopped into 2.5 cm pieces and sent to the AD reactor for methane production. If biochar was included, its production was also analyzed through the LCA model, covering feedstock collection, transportation (50 km), preprocessing, and production. All background processes were based on the Ecoinvent 3 database.^[^ ^46^ ^]^ The LCA model was developed using the openLCA 1.6.3 environmental modeling tool.^[^ ^47^ ^]^ GHG emissions were calculated using 100‐year global warming potentials and converted to CO_2_ equivalents.

### DNA Extraction, Polymerase Chain Reaction, High‐Throughput Sequencing, and Metagenomic Analysis

Digested samples were collected every 4 days, with all samples existing in a mixed solid‐liquid state. After centrifugation, the digestion solids were stored at −80 °C. The digestion liquid was collected at the peak production of the treatments on Day 6 and at the end of gas production on Day 26. In the control, the digestion liquid was collected at peak production on Day 8 and at the end of gas production on Day 26. After centrifugation, microbial were collected for genome DNA extraction and primer design. Bacteria were amplified in the V4/V5 region of the 16S rRNA using the primers 338F (5′‐ACTCCTACGGGAGGCAGCAG‐3′) and 806R (5′‐GGACTACHVGGGTWTCTAAT‐3′). Archaea were amplified using the primers 524F10extF (5′‐TGYCAGCCGCCGCGGTAA‐3′) and Arch958RmodR (5′‐YCCGGCGTTGAVTCCAATT‐3′). The PCR products were subjected to agarose gel electrophoresis and then underwent metagenomic sequencing. After sequencing, the data were processed by splicing, filtering, and quality trimming, with the reads obtained assembled into contigs. Gene prediction was then performed and similarity comparisons made using algorithms to obtain clustered gene sequences. Genes with a nucleic acid length of ≥100 bp were selected and translated into amino acid sequences. The gene sets were compared with the NR, eggNOG, and KEGG databases to obtain annotations for species, COG function, and KEGG pathways, respectively. High throughput sequencing and metagenomic analysis were performed on the Majorbio cloud platform (www.majorbio.com).

### Determination and Analysis Methods

The methods for biochar physicochemical analysis, AD experiments, and energy and payments balance calculations are provided in materials, methods 1–4 and Tables S3 and S4 (Supporting Information). These include the preparation and characterization of modified biochar, physicochemical parameters of substrate and inoculum, VFAs composition determination in AD, bioinformatics, and energy and economic analysis. All experimental conditions in modified biochar, including the AD setup, Raman spectra, and HRTEM, remained consistent with those in the previous experiment involving the AD of maize straw.

### Statistical Analysis

The results were presented as mean ± standard deviation. A *t*‐test was used to analyze the degradation rates of TS and VS. The Kruskal‐Wallis test, followed by post‐hoc Tukey‐Kramer testing, assessed the significance of differences in α diversity indices and KEGG pathway level 3. For other groups, one‐way ANOVA was used, followed by post‐hoc Duncan's test. Significance was determined at p < 0.05.

## Conflict of Interest

The authors declare no conflict of interest.

## Author Contributions

C.Y. and Y.Y. conceived this project. Z.L., M.W., and J.H. processed the AD experiment and collected data. Y.Q., C.Y., X.Z., J.L., Y.W., S.H., D.G., and S.Z. conducted the Preparation of biochar and analyses. CY and YY wrote the first draft of the manuscript, and Z.L., Y.Q., M.W., H.W., H.L., J.H., X.G., X.Y., T.X., and H.C. contributed to subsequent revisions. All authors contributed to the final written product.

## Supporting information



Supporting Information

## Data Availability

Sequence data associated with this project have been deposited in the NCBI Short Read Archive database (Accession Number: PRJNA1251790, PRJNA1252695 and PRJNA1254328).
