# Supplementary material for: Graphitized Biochar Derived from Agricultural Wastes Enhances Methanogenesis via Conductivity‐Driven Direct Interspecies Electron Transfer
Source: Adv Sci (Weinh). 2025 Aug 4;12(41):e08739. doi: 10.1002/advs.202508739 (PMC12591188; doi:10.1002/advs.202508739)
Supplement: Supplementary file 1 — Supporting Information [file ADVS-12-e08739-s001.docx]

**Supplementary Information for**

**Graphitized Biochar Derived from Agricultural Wastes Enhances Methanogenesis via Conductivity-Driven Direct Interspecies Electron Transfer**

**Caiyun Yang^1,3^, ZhenLiu^2,3^, Weiguo Liu^3,5^, Yuxin Qiu^2^,** **Shuai Zhang^6^, Xinke Zhang^2^, Mengyi Wang^2,3^, HengWu^2,3^, Hongyi Lyu^2,7^, Jinzhi Huang^2,8^, Jia Liu^2,3,4^, Yirong Wang^3^, Siying He^2^, Dongze Gu^2^, Xiaohui Guo^2,3,4^, Xuanmin Yang^2,3,4^, Teng Xie^2,3,4^, Heyu Chen^2,3,4^, Yiqing Yao^2,3,4*^,**

*^1^College of Life Science*, *Northwest A&F University*, *Yangling, Shaanxi 712100*, *PR China*;

*^2^College of Mechanical and Electronic Engineering*, *Northwest A&F University*, *Yangling*, *Shaanxi 712100*, *PR China*;

*^3^Interdisciplinary Research Center for Biomass Energy and Materials (IRC-BEM),* *Northwest A&F University*, *Yangling*, *Shaanxi 712100*, *PR China*;

*^4^Northwest A&F University Shenzhen Research Institute, Shenzhen, 518000,China;*

*^5^College of Forestry*, *Northwest A&F University*, *Yangling*, *Shaanxi 712100*, *PR China*;

*^6^College of Food Engineering & Nutritional Science SNNU, Shaanxi Normal University, Xian, Shaanxi 710119*, *PR China*;

^7^*State Key Laboratory of Urtan Water Resource and Environment*, *School of Environment*, *Harbin Institute of Technology*, *Harbin, 150090, PR China*;

^8^*QiCheng Suspension Technology Co,.Ltd, Xian*, *710086*, *China*;

**About the author:**

*Corresponding author.

E-mail addresses: dzhtyao@nwafu.edu.cn; dzhtyao@126.com (Y. Yao)

**Supplementary Materials and Methods**

1. **Preparation method of modified biochar**

Straw and wood feedstocks were cut up approximately 0.5 cm pieces using scissors, while nutshell feedstocks were hammered into approximately 0.5 cm pieces using a hammer. After smashing, the feedstocks were sieved through a 40-mesh sieve and stored in sealed bags for later use. The feedstocks were repeatedly washed with deionized water to remove impurities and then soaked in 1.0 M H₂SO₄ for one day. The feedstocks were then collected, washed, and dried in an electric constant temperature drying oven at 75 °C for 48 hours.

Orthoboric acid and feedstocks powder were evenly dispersed in ultrapure water at various mass ratios, ultrasound for 30 minutes, and then heated with stirring on a magnetic stirrer at 80 °C until the water was completely evaporated. The product was vacuum drying overnight at 90 °C. Obtained mixture was transferred to a crucible and placed in a tube furnace under oxygen-limited conditions. The temperature was increased to 450 °C, 550 °C, and 700 °C at a rate of 5 °C/min, and maintained for 2 hours at each temperature. The biochar was then washed three times with ultrapure water and ethanol, vacuum drying at 60 °C for 12 hours, and ground through a 100-mesh sieve before storage. The content of B in the modified biochar feedstocks is 5%, were labeled and numbered accordingly^[1,2]^.

1. **Characterization method of biochar**

The surface morphology of biochar at 2000x magnification is observed using SEM. The specific surface area is determined using a BET and Langmuir method with a surface area analyzer, and the micropore, mesopore, and macropore sizes are measured with a pore size analyzer. Isothermal nitrogen adsorption and desorption experiments are conducted using the static capacity method, and the adsorption amounts at different relative pressures are recorded. The isotherm data are analyzed based on the BET and Langmuir theories, and the specific surface area is calculated using the BET equation, while the pore size distribution is calculated using the BJH model. The crystal structure of biochar is characterized using XRD, and the functional group composition is analyzed using FTIR. The biochar is passed through an 80-mesh sieve and then mixed uniformly with KBr in a 1:200 mass ratio. The mixture is ground and pressed into a thin pellet to obtain the spectrum^[3]^.

1. **An anaerobic digestion experiment is conducted with biochar addition**

3.1 Determination of total solid: hot air drying

The total solid (TS) content of the sample was determined by the drying method. The crucible was dried to constant weight and the mass of the crucible was recorded as W. The sample filling 1/3 of the volume of the crucible was added to it and the mass of the crucible and the sample was recorded as *W*_1_. The contents were dried at 105 °C for 24 h to constant weight, and the mass of the crucible and the sample was weighed as W_2_. The TS was calculated using the formula:

$$TS=\frac{W_{2}-W}{W_{1}-W}\times100\%$$

3.2 Determination of Volatile solid: high temperature burning method (based on TS)

The volatile solids (VS) content was determined using the high temperature scorch method. The crucible and the sample, which had completed the TS determination, were placed in a muffle furnace, heated up to 550 °C ± 20 °C and kept at that temperature for about 3 h. The instrument was turned off. After the temperature cooled down to below 100 °C, the mass of the crucible and sample *W*3 was weighed. VS was calculated by the formula: .

$$VS=\frac{W_{1}-W_{3}}{W_{1}-W}\times100\%$$

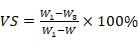


3.3 Determination of gas volume: drainage collecting method

3.4 Determination of gas composition and VFA content

The gas chromatograph was used to determine the gas composition and content, and the reaction conditions were TCD detector inlet temperature 100 ℃, detector temperature 100 ℃, furnace temperature 90 ℃, and the carrier gas was argon at a flow rate of 30 mL/min. The gas chromatograph was used to determine the total acid concentration of VFA, including single fatty acids such as acetic acid, propionic acid, butyric acid, isobutyric acid, valeric acid and isovaleric acid, in the pretreated digestion broth.

1. **Observation of the graphitization structure of biochar and economic benefit analysis**

Nickel foam is used as the support, onto which a mixture of polytetrafluoroethylene and biochar is ground with a small amount of ethanol to form a paste. The paste is evenly applied to the foam, compressed into a sheet, and the biochar electrode is then vacuum-dried. A saturated calomel electrode serves as the reference, while the biochar and platinum sheet electrodes are used as working electrodes to measure the cyclic voltammetry characteristics of the biochar electrode. A dispersive confocal Raman spectrometer analyzes the carbon skeleton structure of biochar. The degree of disorder is determined by the relative intensities of the G and D peaks. The Raman excitation source is a 5 mW, 532 nm laser, with a spectral range of 80 to 4500 cm⁻¹. Three different regions of each biochar sample are tested, and the average ID/IG value, representing the degree of disorder, is calculated^[4]^. HRTEM is conducted with a 100 kV electron beam to locate crystals in the biochar. The biochar is crushed with a mortar and pestle to obtain particles smaller than 100 nm, then dispersed in a small amount of ethanol. The dispersion is ultrasonically treated for 10 to 30 minutes to create a uniform suspension. Two to three drops of the suspension are placed on a copper grid, which is air-dried or oven-dried. The grid is gently blown with an ear bulb to remove loose particles and prevent contamination or damage to the electron microscope. The prepared sample is then observed using HRTEM^[5]^. Dry the carbon material samples at 105 °C for 24 hours and pass them through a 100-mesh sieve to ensure uniform particle size. Accurately weigh 0.2 g of biochar and add it to 50 mL of deionized water into a beaker. The control group was only deionized water, which was used to correct the changes in background pH. Acid titration (determination of alkalinity): Measure the initial pH of the biochar suspension. Add 0.1 M HCl drop by drop (0.1 mL each time), stir for 30 seconds and then record the pH. Titrate to pH 3.0. Record the titration volume and pH changes. Alkaline titration (for acidity determination): Measure the initial pH of the biochar suspension. Add 0.1 M NaOH drop by drop, stir and record pH. Titrate to pH 11.0. Draw the ph-titration volume curve. BC was calculated respectively in different pH ranges to analyze the differences in buffering capacity of biochar at different pH levels.

1. **Theoretical calculation details**

Based on the constructed molecular structure of biochar, the B-doped biochar model is directly loaded, with B at the edge of the biochar^[6]^. When calculating and simulating the adsorption capacity of biochar structure for cytochrome C, if the cytochrome C structure has N/S coordination atoms, the bonding effect between Fe and B cannot be explored. Therefore, only the porphyrin ring of the cytochrome C structure and Fe²⁺ are retained for adsorption simulation. The DFT calculations in this study were performed using the DMol^3^ code in Materials Studio software. The generalized gradient approximation (GGA) with the Perdew-Burke-Ernzerhof (PBE) was used for all calculations. The double numerical plus polarization (DNP) was chosen. Geometric optimization was conducted adopting strict convergence criteria (2 × 10^−5^ Ha for energy, 4 × 10^−3^ Ha Å^−1^ for force, and 5 × 10^−3^ Å for displacement). The self-consistent field convergence criterion of 1 × 10^−5^ Ha was used. The adsorption energy (E_ads_) of FeN4 on the surface of metal sulfides is calculated according to the following equation: E_ads_ = E_total_ − E_FeN4_ − E_metal sulfides_. where E_total_, E_FeN4_, and E_catalyst_ are the total energies of FeN4 on the surface of metal. sulfides, the O_2_ molecule, and metal sulfides, respectively.

1. Environmental impact and economic benefits of adding biochar to AD

This method has been appropriately improved based on the literature calculation methods^[7]^. The calculation of carbon balance theory (g) is based on a relatively state and does not consider trace carbon losses caused by human factors in AD. The total carbon in AD mainly comes from gases (CH_4_, CO_2_), volatile fatty acids (VFAs), and residual solids. The carbon mass of CH_4_ and CO_2_ is calculated based on density under ideal conditions at 25 °C. Energy output (kJ) is primarily come from the heat of combustion of H_2_ and CH_4_, while energy input comes from the electricity consumption of the incubator. The economic benefit (USD) is calculated as follows: the efficiency of converting thermal energy into electrical energy is set at 0.42; the cost of LAB is 27.5 USD/kg; and the electricity price is 0.18 USD/kWh^[7,8]^.

**Supplementary Result**

**
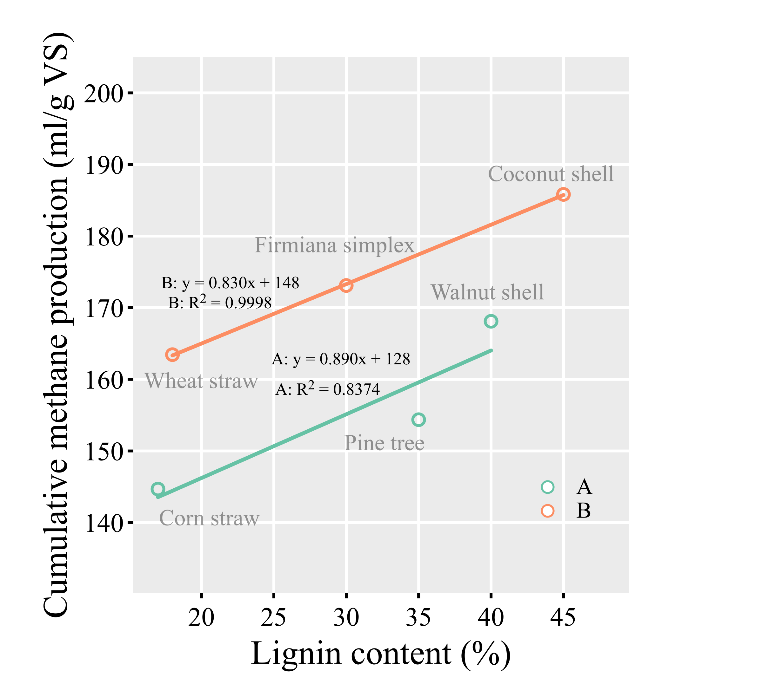
**

**Figure S1:** The correlation between lignin content and cumulative methane production across all feedstocks/temperatures. (The cumulative methane production was the average value of each group. The fitting method was lm and the loess-span was 0.5)


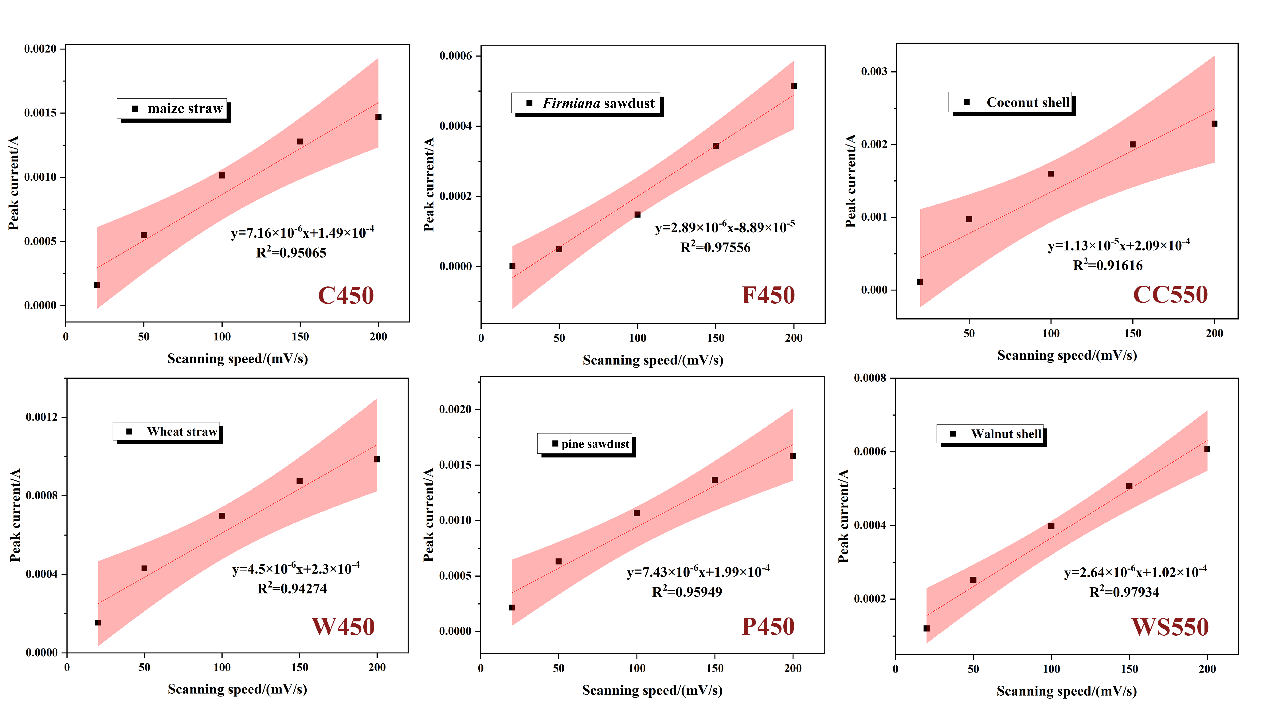


**Figure S2:** the linear relationship between peak current and scanning speed.


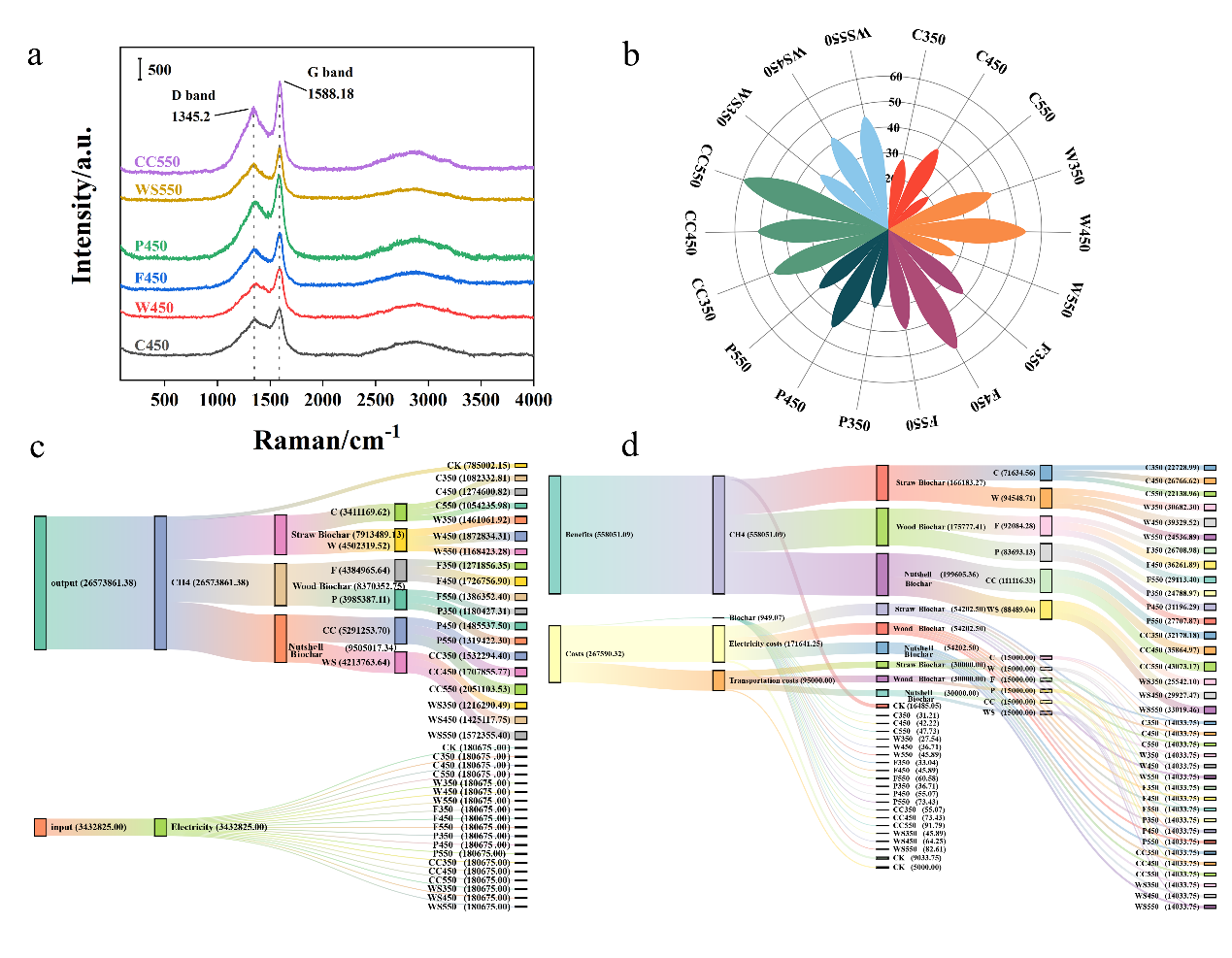


**Figure S3: (a)**Raman spectrum of biochar**(b)** Incremental global warming potential after adding biochar to AD. **(c)** Energy balance calculation. **(d)** Balance of payments calculation.


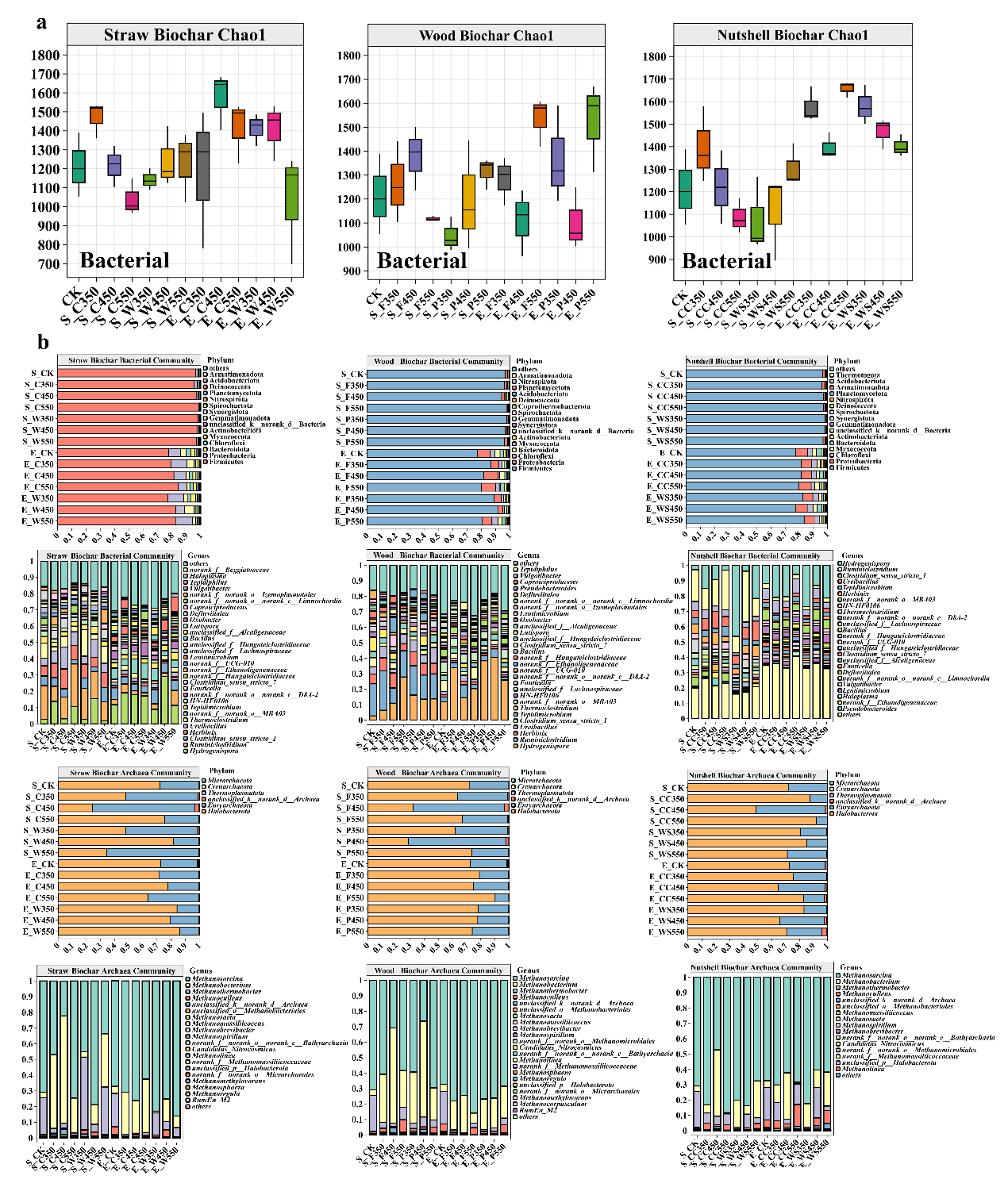


**Fig. S4:** Chao 1 index and community composition of anaerobic digestion microorganisms with the addition of straw, wood, and nutshell biochar. (a) chao 1 index of biochar. (b) Family and genus level community composition of bacterial and archaea with 16SrRNA after biochar addition to AD.


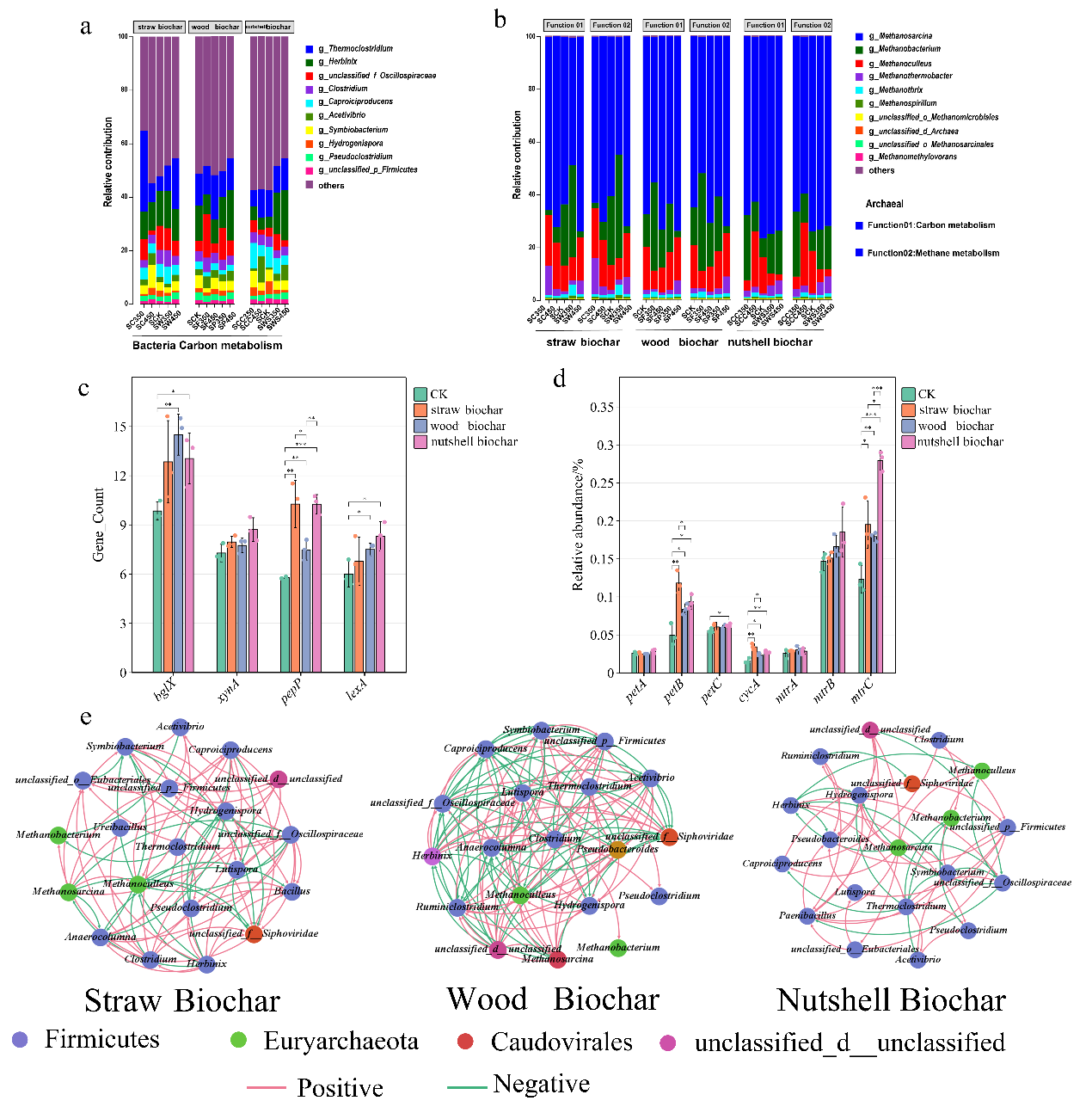


**Fig. S5**: Methane metabolism in AD of maize straw with different biochar. (a) In bacterial carbon metabolism function, the species contribution of the top 10 in total abundance. (b) and in archaea carbon metabolism and methane metabolism function, the species contribution of the top 10 in total abundance. (c) The Gene Count of key genes in protein and hydrolysis phases. (d) Relative abundance/% of key genes in DIET phase. (e) The top 20 bacterial-archaea co-occurrence network in different biochar.

**
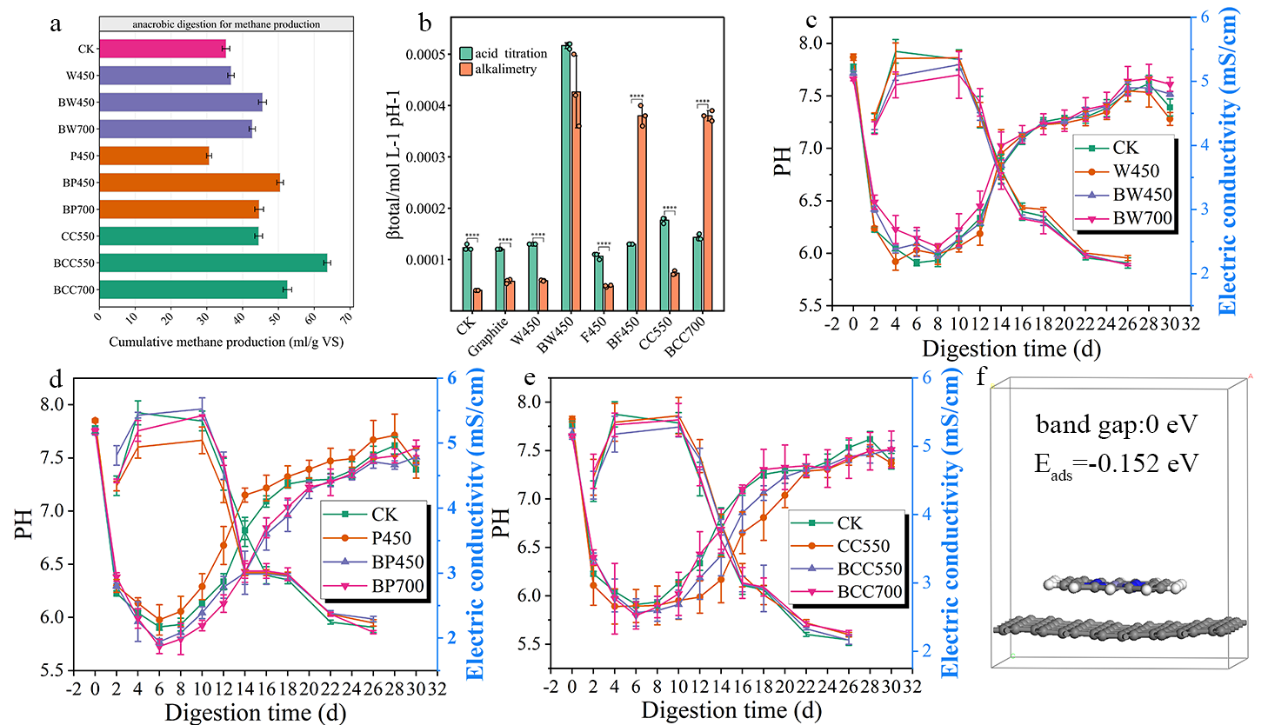
**

**Fig. S6**: Methanogenesis and Conductive characterization of modified biochar. (a) Cumulative methane production in AD with biochar. (b) The acid-base buffering capacity of different carbon materials. (c, d, e) PH, and electrical conductivity for CK, modified biochar. (f) The adsorption energy of cytochrome C by graphite.

**Table S1** Design of biochar preparation test

| **Biochar sample / pyrolysis temperature** | **350℃** | **450℃** | **550℃** |
| --- | --- | --- | --- |
| maize straw biochar | C350 | C450 | C550 |
| Wheat straw biochar | W350 | W450 | W550 |
| *Firmiana* sawdust biochar | F350 | F450 | F550 |
| pine sawdust biochar | P350 | P450 | P550 |
| Walnut shell biochar | WS350 | WS450 | WS550 |
| Coconut shell biochar | CC350 | CC450 | CC550 |

**Table S2** Physicochemical properties of digested feedstocks

|  | **TS（%）** | **VS（%）** | **VS/TS（%）** | | **C（%）** | **N（%）** | **C/N** |
| --- | --- | --- | --- | --- | --- | --- | --- |
| maize straw | 88.85±0.09 | 78.80±0.24 | 88.69±0.18 | 50.02±0.27 | | 0.88±0.04 | 56.84±2.73 |
| sludge | 6.85±0.04 | 3.61±0.03 | 52.73±0.24 | 28.90±0.19 | | 5.26±0.03 | 5.49±0.01 |

**Table S3** Design of modified biochar preparation test

| **Biochar sample / pyrolysis temperature** | **450℃** | **550℃** | **700℃** |
| --- | --- | --- | --- |
| Wheat straw modified biochar | BW450 | - | BW700 |
| pine sawdust modified biochar | BP450 | - | BP700 |
| Coconut shell modified biochar | - | BCC550 | BCC700 |

**Table S4** Physicochemical properties of digested feedstocks in AD of modified biochar

|  | **TS（%）** | **VS（%）** | **VS/TS（%）** | |
| --- | --- | --- | --- | --- |
| maize straw | 90.21±0.03 | 71.25±0.55 | 78.98±0.01 |  |
| sludge | 9.91±0.03 | 5.48±0.30 | 55.32±0.03 |  |

**Table S5** Substrate degradation rate after anaerobic digestion of biochar addition system

| **samples** | **TS degradation rate（%）** | **VS degradation rate（%）** | **samples** | **TS degradation rate（%）** | **VS degradation rate（%）** |
| --- | --- | --- | --- | --- | --- |
| CK | 23.14±0.13 | 29.12±0.02 | - | - | - |
| W350 | 29.67±0.06 | 40.65±0.06 | C350 | 24.95±0.43 | 33.45±0.35 |
| W450 | 40.06±0.54 | 48.86±0.31 | C450 | 27.27±0.24 | 37.71±0.22 |
| W550 | 26.28±0.56 | 34.37±0.67 | C550 | 26.24±0.53 | 35.52±0.49 |
| F350 | 27.43±0.77 | 35.31±0.61 | P350 | 27.89±0.48 | 35.85±0.42 |
| F450 | 32.12±0.23 | 42.25±0.13 | P450 | 34.06±0.28 | 41.66±0.23 |
| F550 | 28.18±0.42 | 36.70±0.36 | P550 | 30.08±0.12 | 39.19±0.08 |
| WS350 | 27.19±0.06 | 34.51±0.21 | CC350 | 29.46±0.53 | 39.09±0.03 |
| WS450 | 29.82±0.56 | 38.31±0.20 | CC450 | 30.63±0.43 | 41.72±0.17 |
| WS550 | 32.90±0.54 | 40.41±0.14 | CC550 | 39.02±0.24 | 47.75±0.20 |

**Table S6**. Specific surface area pore volume and pore size of Biochar

| **Samples** | **SSA (m^2^/g)** | | **Total pore volume (m^3^/g)** | **Average pore diameter (nm)** | **Total microporous pore volume (m^3^/g**  **×10^-3^)** | **Micropore median pore size (nm)** |
| --- | --- | --- | --- | --- | --- | --- |
|  | **BET** | **Langmuir** |  |  |  |  |
| C350 | 5.06±0.22 | 2.92±0.14 | 0.07±0.00 | 39.06±4.55 | 2.12±0.13 | 1.24±0.09 |
| C450 | 7.41±0.24 | 4.08±0.22 | 0.11±0.01 | 15.59±1.01 | 3.08±0.17 | 1.20±0.07 |
| C550 | 7.36±0.19 | 4.07±0.14 | 0.09±0.00 | 17.91±1.14 | 2.93±0.19 | 1.20±0.09 |
| W350 | 9.95±0.64 | 6.26±0.23 | 0.11±0.00 | 33.35±3.82 | 4.39±0.29 | 1.40±0.13 |
| W450 | 14.95±0.89 | 7.22±0.25 | 0.16±0.02 | 11.72±1.05 | 5.04±0.47 | 1.16±0.08 |
| W550 | 11.43±0.71 | 7.21±0.21 | 0.14±0.01 | 15.81±3.95 | 4.43±0.36 | 1.21±0.10 |
| F350 | 7.54±0.19 | 3.81±0.21 | 0.10±0.01 | 38.99±4.51 | 3.13±0.24 | 1.41±0.12 |
| F450 | 10.16±0.54 | 6.87±0.52 | 0.16±0.02 | 24.05±1.01 | 5.31±0.47 | 1.12±0.06 |
| F550 | 8.71±0.32 | 5.58±0.64 | 0.15±0.02 | 26.95±3.28 | 3.09±0.32 | 1.35±0.15 |
| P350 | 4.85±0.02 | 2.38±0.01 | 0.01±0.00 | 27.52±2.99 | 0.11±0.00 | 1.40±0.14 |
| P450 | 8.85±0.49 | 7.78±0.77 | 0.24±0.02 | 21.77±1.01 | 5.43±0.33 | 1.26±0.08 |
| P550 | 5.22±0.16 | 3.68±0.09 | 0.06±0.00 | 23.38±2.36 | 2.42±0.14 | 1.32±0.13 |
| CC350 | 16.73±0.37 | 6.95±0.17 | 0.05±0.00 | 38.43±4.57 | 3.14±0.19 | 1.25±0.06 |
| CC450 | 17.10±0.54 | 7.34±0.22 | 0.10±0.01 | 35.02±4.19 | 3.28±0.24 | 1.17±0.08 |
| CC550 | 17.11±0.48 | 7.79±0.23 | 0.11±0.01 | 17.76±1.01 | 3.31±0.11 | 1.14±0.05 |
| WS350 | 13.32±0.24 | 4.98±0.09 | 0.05±0.00 | 35.40±3.51 | 1.35±0.15 | 1.28±0.09 |
| WS450 | 14.26±0.31 | 5.60±0.11 | 0.06±0.00 | 35.40±5.26 | 2.39±0.17 | 1.23±0.10 |
| WS550 | 14.37±0.28 | 5.70±0.15 | 0.06±0.00 | 27.97±2.17 | 2.46±0.26 | 1.19±0.07 |

***SSA**: specific surface area, **Total pore volume**: Total pore volume of mesopore and macropore, **Average pore diameter:** Average pore size of mesopores and macropores

**Table S7** Archaeal diversity index of anaerobic digestion system with biochar addition

| **Samples** | **Shannon** | **Simpson** | **Ace** | **Chao1** | **Coverage（%）** |
| --- | --- | --- | --- | --- | --- |
| S_CK | 3.44±0.05 | 0.09±0.01 | 1075.25±201.48 | 1014.77±168.13 | 99.54±0.08 |
| E_CK | 4.28±0.11 | 0.08±0.00 | 1128.58±289.76 | 1325.69±149.65 | 99.17±0.04 |
| S_C350 | 4.05±0.27 | 0.05±0.02 | 1425.56±85.77 | 1216.88±107.58 | 99.12±0.07 |
| S_C450 | 4.12±0.16 | 0.05±0.00 | 1426.88±102.78 | 1469.87±94.96 | 99.17±0.04 |
| S_C550 | 3.85±0.05 | 0.08±0.00 | 1198.00±181.74 | 1040.70±96.83 | 99.23±0.03 |
| E_C350 | 4.80±0.10 | 0.03±0.00 | 1460.28±369.45 | 1488.17±367.57 | 99.38±0.04 |
| E_C450 | 4.85±0.30 | 0.03±0.01 | 1586.90±79.27 | 1576.67±150.62 | 99.19±0.07 |
| E_C550 | 4.46±0.16 | 0.05±0.01 | 1218.64±149.82 | 1416.12±162.78 | 99.28±0.03 |
| S_W350 | 3.89±0.23 | 0.06±0.02 | 1330.41±120.40 | 1143.17±56.00 | 99.35±0.04 |
| S_W450 | 4.16±0.29 | 0.06±0.03 | 1481.14±267.08 | 1245.17±157.65 | 99.31±0.11 |
| S_W550 | 3.83±0.02 | 0.07±0.01 | 1370.69±176.68 | 1231.09±184.58 | 99.27±0.09 |
| E_W350 | 4.32±0.67 | 0.04±0.02 | 1442.26±53.36 | 1412.52±83.65 | 99.36±0.12 |
| E_W450 | 4.80±0.12 | 0.03±0.00 | 1483.16±175.85 | 1435.74±259.62 | 99.41±0.15 |
| E_W550 | 4.30±0.70 | 0.06±0.05 | 1422.61±234.19 | 1408.97±150.76 | 99.55±0.07 |
| S_F350 | 3.85±0.05 | 0.08±0.01 | 1198.00±181.74 | 1114.77±168.13 | 99.31±0.07 |
| S_F450 | 4.13±0.05 | 0.05±0.01 | 1426.88±102.78 | 1246.88±107.53 | 99.56±0.04 |
| S_F550 | 4.05±0.27 | 0.05±0.00 | 1375.25±201.48 | 1210.70±96.83 | 99.23±0.03 |
| E_F350 | 4.46±0.16 | 0.05±0.00 | 1160.28±369.45 | 1188.17±367.57 | 99.38±0.04 |
| E_F450 | 4.85±0.10 | 0.03±0.00 | 1586.90±79.27 | 1576.67±150.62 | 99.15±0.07 |
| E_F550 | 4.80±0.30 | 0.03±0.01 | 1418.64±149.82 | 1416.12±162.78 | 99.28±0.03 |
| S_P350 | 3.83±0.23 | 0.07±0.01 | 1330.70±176.70 | 1131.09±184.58 | 99.37±0.04 |
| S_P450 | 4.16±0.29 | 0.06±0.03 | 1481.14±267.08 | 1245.17±157.65 | 99.31±0.11 |
| S_P550 | 3.89±0.02 | 0.06±0.02 | 1370.41±120.40 | 1243.17±56.00 | 99.27±0.09 |
| E_P350 | 4.30±0.70 | 0.08±0.01 | 1352.61±234.19 | 1335.74±295.62 | 99.36±0.12 |
| E_P450 | 4.80±0.12 | 0.03±0.00 | 1483.16±175.85 | 1412.52±83.65 | 99.41±0.15 |
| E_P550 | 4.32±0.67 | 0.06±0.05 | 1442.26±53.36 | 1408.97±150.76 | 99.53±0.07 |
| S_CC350 | 4.13±0.20 | 0.06±0.00 | 1166.48±115.12 | 1087.83±77.71 | 99.23±0.07 |
| S_CC450 | 4.21±0.19 | 0.05±0.01 | 1379.50±279.35 | 1220.74±162.40 | 99.18±0.04 |
| S_CC550 | 4.21±0.09 | 0.05±0.02 | 1437.20±170.40 | 1396.56±167.78 | 99.57±0.03 |
| E_CC350 | 4.73±0.30 | 0.04±0.02 | 1371.68±52.24 | 1397.41±57.89 | 99.38±0.04 |
| E_CC450 | 4.76±0.29 | 0.04±0.03 | 1643.54±12.27 | 1576.58±78.85 | 99.19±0.07 |
| E_CC550 | 5.02±0.03 | 0.02±0.00 | 1724.72±209.41 | 1657.25±34.40 | 99.20±0.03 |
| S_WS350 | 3.46±0.53 | 0.08±0.01 | 1133.98±277.96 | 1075.94±165.89 | 99.44±0.04 |
| S_WS450 | 3.81±0.23 | 0.09±0.04 | 1192.54±110.83 | 1113.94±189.60 | 99.61±0.11 |
| S_WS550 | 3.91±0.39 | 0.08±0.00 | 1293.01±303.57 | 1306.63±93.96 | 99.90±0.09 |
| E_WS350 | 4.61±0.03 | 0.05±0.00 | 1412.95±14.75 | 1401.46±47.91 | 99.36±0.12 |
| E_WS450 | 4.74±0.23 | 0.04±0.02 | 1530.18±200.36 | 1465.76±68.04 | 99.47±0.15 |
| E_WS550 | 4.87±0.06 | 0.02±0.00 | 1650.87±149.47 | 1581.58±87.31 | 99.55±0.07 |

**Table S8** Molecular docking results of cellulose and *bglX*-encoding enzyme

| mode | affinity | dist from best mode | |
| --- | --- | --- | --- |
|  | (kcal/mol) | rmsd l.b. | rmsd u.b. |
| 1 | -6.7 | 0 | 0 |
| 2 | -6.5 | 2.045 | 6.034 |
| 3 | -6.5 | 1.836 | 3.284 |
| 4 | -6.3 | 21.126 | 23.779 |
| 5 | -6.3 | 21.667 | 24.336 |

**Table S9** Molecular docking results of hemicellulose and *xynA* -encoding enzyme

| mode | affinity | dist from best mode | |
| --- | --- | --- | --- |
|  | (kcal/mol) | rmsd l.b. | rmsd u.b. |
| 1 | -5.5 | 0 | 0 |
| 2 | -5.0 | 0.718 | 2.867 |
| 3 | -4.6 | 24.020 | 25.356 |
| 4 | -4.3 | 1.572 | 2.221 |
| 5 | -4.1 | 26.183 | 27.159 |

**References**

[1] B. Liu, W. Guo, H. Wang, Q. Si, Q. Zhao, H. Luo, N. Ren, *Chem. Eng. J.* **2020**, *396*, 125119.

[2] S. Gao, Z. Wang, H. Wang, Y. Jia, N. Xu, X. Wang, J. Wang, C. Zhang, T. Tian, W. Shen, *Appl. Surf. Sci.* **2022**, *599*, 153917.

[3] X. H. Dai, H. X. Fan, C. Y. Yi, B. Dong, S. J. Yuan, *J. Mater. Chem. A* **2019**, *7*, 6849.

[4] C. Chen, K. Sun, C. Huang, M. Yang, M. Fan, A. Wang, G. Zhang, B. Li, J. Jiang, W. Xu, J. Liu, *Biochar* **2023**, *5*.

[5] F. Destyorini, S. Priyono, H. S. Oktaviano, Y. I. Hsu, R. Yudianti, H. Uyama, *Waste and Biomass Valorization* **2024**, *15*, 2881.

[6] H. Xie, H. Ao, L. Xu, S. Ao, T. Zhang, W. Li, Y. Yang, *Biomass Convers. Biorefinery* **2024**, *14*, 13547.

[7] H. Wu, H. Zhang, R. Yan, S. Li, X. Guo, L. Qiu, Y. Yao, *Adv. Sci.* **2024**, *11*, 2406119.

[8] F. Demichelis, T. Tommasi, F. A. Deorsola, D. Marchisio, G. Mancini, D. Fino, *Chemosphere* **2022**, *289*, 133058.
